# Psychopathological Burden among Healthcare Workers during the COVID-19 Pandemic Compared to the Pre-Pandemic Period

**DOI:** 10.3390/ijerph20247153

**Published:** 2023-12-07

**Authors:** Sara Gostoli, Angelica D’Oronzo, Carlotta Malaguti, Francesco Guolo, Cristian Balducci, Regina Subach, Vittorio Lodi, Carmine Petio, Chiara Rafanelli

**Affiliations:** 1 Department of Psychology “Renzo Canestrari”, University of Bologna, 40127 Bologna, Italy; sara.gostoli2@unibo.it (S.G.); angelica.doronzo@studio.unibo.it (A.D.); regina.subach2@unibo.it (R.S.); chiara.rafanelli@unibo.it (C.R.); 2 Occupational Health Unit, Bologna University Hospital Authority St. Orsola Malpighi Polyclinic IRCCS, 40138 Bologna, Italy; carlotta.malaguti@studio.unibo.it (C.M.); vittorio.lodi@aosp.bo.it (V.L.); 3 Division of Cardiology, Bellaria Hospital, AUSL Bologna, 40139 Bologna, Italy; francesco.guolo@studio.unibo.it; 4 Department of Quality of Life Sciences, University of Bologna, 47921 Rimini, Italy; cristian.balducci3@unibo.it; 5 Department of Psychiatry, Bologna University Hospital Authority St. Orsola Malpighi Polyclinic IRCCS, 40138 Bologna, Italy

**Keywords:** COVID-19, healthcare workers, occupational medicine, psychiatric diagnosis, psychopathological burden

## Abstract

This retrospective observational study on hospital staff requesting an “application visit” (from 2017 to 2022) at the Occupational Medicine department aimed at comparing a “pre-COVID group” (2017–2019) with a “COVID group” (2020–2022) regarding (a) sociodemographic data (i.e., age, sex, occupation, years of employment at the hospital), (b) rate and type of psychiatric diagnoses in both groups and rate of psychiatric diagnoses per subject, and (c) rate of drug/psychotherapeutic prescriptions. Two hundred and five healthcare workers (F = 73.7%; mean age = 50.7 ± 10.33) were visited. Compared with the pre-COVID group, healthcare workers evaluated during COVID-19 were significantly younger and reported fewer years of employment at the hospital. Although rates of primary psychiatric diagnoses were similar in both samples, an increased number of psychopathologies per subject and associated treatment prescriptions in the COVID group was observed. In the COVID group, 61% had one psychiatric diagnosis, and 28% had 2+ psychiatric diagnoses, compared with 83.8% and 6.7% of pre-COVID. Furthermore, 56.2%/1.9% in pre-COVID and 73%/6% in the COVID group were prescribed drugs/psychotherapy, respectively. The findings of the present study highlighted an increase in both younger workers’ requests and psychiatric comorbidities during the pandemic, representing a burden on the Italian healthcare system. It is thus relevant to address the mental health challenges of healthcare workers accordingly.

## 1. Introduction

Throughout 2020, healthcare professionals faced an extraordinary situation due to the COVID-19 pandemic, a newly emerged and poorly understood disease characterized by a high mortality rate among at-risk patients [1]. During the pandemic, healthcare workers in various medical specialties reported an exacerbation in stress and a decrease in their quality of life [2]. Recent literature highlighted the pandemic impact on healthcare professionals’ mental health, especially for those on the frontline [3,4]. In particular, anxiety, depression [5,6], and post-traumatic stress disorders [7], without focusing on possible comorbidities, were detected. Healthcare personnel, particularly nurses, were daily exposed to the risk of contracting the virus and infecting their family members, further increasing anxiety levels [8,9]. These concerns were observed especially in Europe [10,11] and particularly in Italy, compared to Chinese healthcare personnel [12]. Occupational Medicine took this extraordinary situation as an opportunity to highlight the importance of its role thanks to both the implementation of prevention measures at the workplace [13] to protect workers’ well-being [14] and emphasis on the rehabilitation of healthcare personnel focusing on their physical health subsequent to viral exposure [15,16]. In a recent study conducted in the pre-pandemic period [17], psychopathological concerns were observed among healthcare professionals who requested medical evaluation from the Occupational Medicine department. However, the primary focus of the mentioned study was to explore job limitations assignments according to psychiatric diagnoses [17]. To the best of our knowledge, there are no studies evaluating healthcare workers’ first visit (i.e., application visit) requests to the Occupational Medicine department, as well as their underlying psychopathological conditions before and after COVID-19. The purpose of the present study is based on the hypothesis that, following the onset of the pandemic in comparison with the pre-pandemic period, healthcare workers’ psychopathological burden, including psychiatric diagnoses and consequent treatments, was generally increased and spread among healthcare workers, thus enhancing the number of application visits related to psychopathological concerns. Therefore, the objective of the present observational investigation, which refers to the three years before COVID-19 pandemic (2017–2019; “pre-COVID group”) and the three years during COVID-19 pandemic (2020–2022; “COVID group”), was to compare “pre-COVID group” with “COVID group” regarding: (a) sociodemographic data (i.e., age, sex, occupation, years of employment at hospital), (b) rate and type of psychiatric diagnoses in both groups and rate of psychiatric diagnoses per subject, and (c) rate of drug/psychotherapeutic prescriptions.

## 2. Materials and Methods

### 2.1. Sample

All the consecutive first visits, the so-called “application visits” (as established by the Italian law regarding workers’ health), at the Department of Occupational Medicine, requested in the period between January 2017 and December 2022 (*N* = 205), were considered. As described in a previous publication [17], all workers asking for an application visit were evaluated first by the occupational physician for any orthopedic limitations and, subsequently, by the occupational psychiatrist for any psychiatric limitations. Thereafter, all the subjects received a medical certificate based on their physical and mental health status, as follows: fit for work, unfit for work, and fit with limitations. The allocation into two sub-groups was based on the year of the visit, where the “pre-COVID group” (*N* = 105) referred to the period from January 2017 to December 2019 and the “COVID group” (*N* = 100) to the period from January 2020 to December 2022.

### 2.2. Assessment

The sample data were retrospectively examined from September 2022 to April 2023 by one of the clinical psychologists who collaborated in the study, using the web platform Infoclin for data up to September 2022 and subsequently with another platform called Arianna Portal (version 2303.01.05.00; Dedalus Healthcare System Group, Florence, Italy), the software used for managing clinical health data at the University Hospital. Sociodemographic information regarding age, sex, occupation, and years of employment at the hospital was collected. Additionally, data concerning the year of the application visit, diagnoses of mental disorders according to DSM-5 [18], and pharmacological and/or psychotherapeutic treatment were reported. The information underwent anonymous analysis in accordance with the Italian data protection legislation (Legislative Decree No. 196/2003). The research was carried out following the ethical guidelines outlined in the Declaration of Helsinki [19].

### 2.3. Data Analysis

Descriptive data analyses for the present study covering the period from January 2017 to December 2022 were carried out. A Chi-square test was conducted to examine the relationship between the “group” variable, divided into “pre-COVID group” (2017–2019) and “COVID group” (2020–2022), and the “diagnostic category” variable, classified as DSM-5 [18] “substance-use disorders”, “feeding and eating disorders”, “anxiety disorders”, “sleep–wake disorders”, “trauma and stressor-related disorders”, “mood + bipolar disorders”, “obsessive–compulsive and related disorders”, “none”, “personality disorders”, “somatic symptoms and related disorders” and “schizophrenia spectrum and other psychotic disorders”. Burnout syndrome was included in the “trauma and stressor-related disorders” category. A Chi-square test was conducted to examine the relationship between “group” (i.e., “pre-COVID group” 2017–2019 versus “COVID group” 2020–2022) and “number of psychiatric diagnoses” variable, categorized as “no diagnosis”, “one diagnosis” and “2+ diagnoses” per subject. In addition, a logistic regression model was used to investigate the possible effect between “group” and “number of psychiatric diagnoses” variables, the latter being re-coded into two levels (i.e., “no diagnosis” and “1+ diagnoses”), adding age and sex as covariates. Finally, a Chi-square test was conducted to examine the relationship between the “group” and “prescriptions” variable, which was categorized as “none”, “medication”, and “psychotherapy”. Also, a logistic regression model was used to investigate the possible effect between “group” and “prescriptions” variables, the latter being re-coded into two levels (i.e., “no prescription” and “medications/psychotherapy”), adding age and sex as covariates. If the *p*-values were found to be non-significant (>0.05), it would indicate an absence of a relationship between the two variables. Conversely, if they were significant (<0.05), it would signify a relationship between the two variables.

## 3. Results

### 3.1. Description of the Sample

The overall sample (*N* = 205) (i.e., healthcare workers evaluated from January 2017 to December 2022) had a mean age of 50.7 years (SD = 10.3) and included mainly females (*N* = 151, 73.7%) and nurses (*N* = 96, 46.8%). The mean years of employment at the hospital was 18.9 (SD = 11.1). The pre-COVID group (*N* = 105) had a mean age of 53.9 years (SD = 9.2), ranging from 29 to 69 years, and included mainly females (*N* = 78, 74.3%) and nurses (*N* = 51, 48.7%). The mean years of employment at the hospital was 22.5 (SD = 10.5). The COVID group (*N* = 100) had a mean age of 47.3 years (SD = 10.4), ranging from 25 to 65 years, and included mainly females (*N* = 73, 73%) and nurses (*N* = 45, 45%), as well. The mean years of employment at the hospital was 11.2 (SD = 10.6). See Table 1 for data completeness.

Among the 205 healthcare workers, 184 (89.8% of the total sample) received a psychiatric diagnosis. Among them, 149 (81%) subjects received one diagnosis; 35 subjects (19%) received a primary diagnosis with the concurrent presence of at least one other DSM-5 disorder. Among these subjects, 28 (15.2%) received two diagnoses, whereas 7 (3.8%) received three diagnoses. In total, 224 diagnoses were issued by the occupational psychiatrist, with an average of 1.09 (diagnoses) per individual. Ten different types of diagnostic categories were issued (see Table 2), considering the DSM-5 [18] specific diagnostic area of the primary diagnosis and disregarding comorbidities. Regarding the first diagnoses grouped into categories (excluding comorbidities) according to the DSM-5 [18], 68.5% of healthcare workers presented with mood and bipolar disorders. In particular, 28.8% presented with depressive disorder, 11.4% anxious–depressive disorder, 10.3% reactive depressive disorder, and 3.8% bipolar disorder. Other common disorders included trauma and stress-related disorders, accounting for 12.5%, in particular adjustment disorders with depressed mood (3.8%). Furthermore, 9.2% of healthcare workers presented anxiety-related disorders, whereas 2.7% had substance-use disorders. See Table 2 and Table A1 for data completeness.

In addition, among the 184 healthcare workers affected by a psychiatric disorder, the occupational psychiatrist prescribed pharmacological therapy in 71.7% and psychotherapy in 4.3% of the cases. A total of 35.3% did not require any pharmacological or psychotherapeutic prescription (Table 3).

### 3.2. Comparison between “Pre-COVID Group” and “COVID Group” Regarding Sociodemographic Data

Concerning sociodemographic variables, compared with healthcare workers belonging to the pre-COVID group, those who requested an application visit during COVID were significantly younger (pre-COVID group mean age = 53.9 ± 9.2 years; COVID group mean age = 47.3 ± 10.4 years; F = 22.942, *p* < 0.001) and had fewer years of employment at the hospital (pre-COVID group mean years of employment = 22.5 ± 10.5; COVID group mean years of employment = 15.2 ± 10.6; F = 24.269, *p* < 0.001). On the contrary, no significant difference in sex and occupation was found (*p* > 0.05).

### 3.3. Comparison between “Pre-COVID Group” and “COVID Group” Regarding Diagnostic Categories

In general, the rates regarding the type of primary psychiatric diagnoses were similar in both groups (i.e., no statistical difference was detected). The only qualitative difference referred to the diagnoses of trauma and stress-related disorders. Indeed, in the pre-COVID group, they were reported in 7.6% of the subjects, whereas in the COVID group, they were reported in 15%. However, this difference did not reach statistical significance. See Table 4 for data completeness.

### 3.4. Comparison between “Pre-COVID Group” and “COVID Group” Regarding Diagnoses Rate

Concerning the rate of psychiatric comorbidities, a significant difference between pre-COVID and COVID groups has been observed (Table 5). In terms of the mean number of diagnoses per subject, there was a significant increase in the COVID group (mean number of diagnoses per subject in the pre-COVID group = 0.97, in the COVID group = 1.22; F = 9.723, *p* < 0.002). In the pre-COVID group, out of a total of 105 healthcare workers, 95 subjects (90.5%) received at least one psychopathological diagnosis. Among these, 88 (92.6%) received only one diagnosis, and 7 (7.3%) received two diagnoses. In the COVID group, out of a total of 100 healthcare workers, 89 (89%) received at least one psychopathological diagnosis. Among them, 61 (68.5%) received one diagnosis, 23 (25.8%) received two diagnoses and 5 (5.6%) received three diagnoses. Healthcare workers who received only one diagnosis were 83.8% in the pre-COVID group and 61% in the COVID group. Conversely, 6.7% in the pre-COVID group and 28% in the COVID group reported two or more diagnoses. An increase for the “2+ diagnoses” category and a decrease for the “1 diagnosis” category from the pre-COVID group to the COVID group was thus detected. As previously observed, primary diagnostic categories did not change among healthcare workers. Furthermore, in the COVID group triple diagnoses, a phenomenon that was not present in the pre-COVID group, occurred. See Table 5 for data completeness. Regression analysis showed that compared with the pre-COVID group, the COVID group had a significantly higher probability of having one or more DSM-5 diagnoses (versus no diagnosis) (Odd Ratio [OR] = 5.35; 95% Confidence Interval [CI]: 2.15–13.36; *p* < 0.001) when adjusted for age and sex.

### 3.5. Comparison between “Pre-COVID Group” and “COVID Group” Regarding Prescriptions

A significant increase in both pharmacological and psychotherapeutic prescriptions and a decrease in the absence of any therapy were found (Table 6). In particular, regarding pharmacological prescriptions, these were made in 56.2% of the cases in the pre-COVID group and 73% of the cases in the COVID group. Concerning psychotherapy prescriptions, the rate was 1.9% in the pre-COVID group and 6% in the COVID group. A total of 41.9% of subjects in the pre-COVID group and 21% in the COVID group did not receive any prescription. Regression analysis showed that, compared with the pre-COVID group, the COVID group had a significantly higher probability of having a prescription of medications/psychotherapy (versus no prescription) (OR = 3.11; 95%CI: 1.60–6.06; *p* = 0.001), when adjusted for age and sex.

## 4. Discussion

To the best of our knowledge, the current study represents the first attempt to analyze healthcare workers’ first application visits before (from January 2017 to December 2019) and during the COVID-19 pandemic (from January 2020 to December 2022). In particular, the objective of the present investigation was to compare the “pre-COVID group” with the “COVID group” regarding sociodemographic data (i.e., age, sex, occupation, and years of employment at the hospital), rate/type of psychiatric diagnoses according to DSM-5 in both groups and rate of psychiatric diagnoses per subject, and rate of drug/psychotherapeutic prescriptions.

Initially, descriptive analyses revealed that the majority of requests came from women and nurses, as in the previous research [17]. In the general population, women present the highest number of psychiatric illness because of biological, social, cultural and psychological factors [20]. Indeed, they are more vulnerable to developing depressive disorders [21] or anxiety symptoms [22] than men. Furthermore, it was reported that women are more likely to consult a general practitioner and seek help for mental health issues compared to men, in the general population [23]. Moreover, in the present findings, among healthcare workers, nurses were more likely to request an application visit to the Occupational Medicine department, probably due to the nature of their work, increased high levels of job-related stress, low job satisfaction, and health issues [24]. In addition, the pandemic and its consequences acted as a risk factor contributing to elevated levels of anxiety, stress, and depression [25,26]. This was demonstrated by a study conducted in two hospitals in Singapore, where nurses reported high levels of anxiety, depression, and PTSD [27] due to challenges faced by healthcare staff [28]. Furthermore, difficult decisions were made, such as choosing which patients have access to life support and which do not [29].

In the present study, DSM-5 psychiatric diagnoses among healthcare professionals were heterogeneous, and no relationships were found between different diagnostic areas and the two groups. Healthcare workers’ job complexity existed even before the onset of the pandemic. This is consistent with previous research that identified numerous psychiatric disorders affecting this category of workers [30,31], especially nurses [32]. For this category, indeed, it is likely that any form of error is ideally not tolerated [33]. Prior to the onset of the COVID-19 pandemic, there were elevated levels of reported depression and anxiety [34]. Our findings align with this pattern, as our pre-COVID data also indicated a high prevalence of diagnoses related to mood disorders in the two groups. As a matter of fact, in previous research, the most common diagnosis was depressive disorder [17], whereas in the present study, there was an increase in diagnoses related to trauma and stress factors. In the pre-COVID group, these were present at a rate of 7.6%, while in the COVID group, the rate was 15%. There was an escalation in diagnoses of adjustment disorders, with anxious and/or depressive symptomatology and also diagnoses of work-related stress. This is emphasized, in line with our results, in the study conducted by Preti and colleagues [35], referring to work-related stress exacerbated by the pandemic, which also contributes to a decrease in job satisfaction [36]. Prolonged exposure to stress can endanger an individual’s homeostatic system, causing a biological imbalance [24]. This is connected to the concept of allostatic load [37,38,39], which refers to everyday occurrences and major challenges defined as life events. When environmental challenges exceed an individual’s capacity to cope, allostatic overload occurs [40,41]. The latter was found to be associated with depressive and anxious symptoms [42,43] but also with high levels of psychological distress [44]. Associations between work-related stress and allostatic load were also found [45].

Regarding differences between healthcare workers who requested a first application visit before or during the COVID-19 pandemic, the present study showed that the latter were significantly younger and reported fewer years of employment at the hospital. In the same vein, the literature showed that the prevalence of mental disorders was significantly higher among healthcare workers of younger age during the first wave of the pandemic [46]. Furthermore, it was observed that the odds of depression [47] and anxiety [47,48] were significantly higher among younger healthcare workers and that newly hired personnel were more likely to be subjected to anxiety and work-related stress [49]. As this group of workers is experiencing the most significant impact due to the pandemic, it is appropriate for hospitals to prioritize prevention and rehabilitation efforts designed for this group of healthcare professionals. 

As for the comparison of the “diagnostic rate” between the two groups, it was observed that in the pre-COVID group, only 6.7% of healthcare workers received two or more coexisting psychiatric diagnoses, while in the COVID group, the rate was 28%. In the present study, the higher rate of comorbidities per subject in the COVID group in comparison with the pre-COVID one is quite impactful from a public health perspective. The COVID-19 pandemic disproportionately affected healthcare personnel, especially frontline physicians [50,51]. Indeed, in the literature, psychiatric comorbidities were identified as the main risk factor for suicide among physicians [52,53]. In particular, in the study conducted by Iannelli and colleagues [54] regarding suicidal behavior among physicians, the primary diagnosis consisted of mood disorders with comorbid substance-use disorders. In line with these results, in the present study, the most frequent primary diagnoses made by the occupational psychiatrist were unipolar and bipolar mood disorders (grouped into a single category), disorders related to traumatic and stressful events (including burnout), and anxiety disorders. Comorbidities associated with these three categories mainly involved sleep–wake, alcohol use, and panic disorders (see Table A1 in Appendix A for data completeness).

In addition, in the pre-COVID group, the percentage of drug prescriptions was 56.2%, while in the COVID group, it rose to 73%. This, on the one hand, could be the consequence of the higher frequencies of multimorbid cases. However, on the other hand, polypharmacy may complicate patients’ symptomatology. Indeed, severe side effects of antidepressants and anxiolytics include headaches, sexual dysfunction, addiction, seizures, and suicide [55]. Moreover, prolonged use of benzodiazepines is associated with worse outcomes in substance-use disorders and comorbid mental disorders [56]. Another important finding of the present study was the increase in the prescription of psychotherapies, rising from 1.9% in the pre-COVID group to 6% in the COVID group. This may be connected to an integrated therapeutic approach that includes both pharmacotherapy and psychotherapy, which is the most appropriate for addressing complex psychiatric comorbidities [57].

This study aimed to provide an overview of the conditions of healthcare workers, focusing on a sample from a University Hospital in Northern Italy. It highlighted an increase in comorbidities among diagnoses related to healthcare workers. If the psychiatric conditions are not properly treated, this could represent a potential risk to both patient safety and the healthcare system [58]. Occupational Medicine should then implement preventive and intervention measures since it is crucial not to focus solely on individual healthcare workers [59] but rather to adopt measures aimed at preserving the integrity of the entire Italian healthcare system, which was severely impacted by the COVID-19 pandemic and was inadequately prepared [12]. A study conducted at Verona University Hospital in Italy [12] highlighted that healthcare workers faced stressful working conditions due to their sudden reassignment to different hospital units or new, unfamiliar tasks. Additionally, it is hypothesized that healthcare workers may have developed psychopathological symptoms as a direct response to the pandemic, without pre-existing conditions, since only 6% of healthcare workers had reported suffering from any disorder before the pandemic [12]. More research needs to be conducted in other Italian hospitals to obtain a comprehensive view of the national situation. In this context, occupational medicine plays a crucial role and should actively engage in safeguarding the health of healthcare workers, especially the vulnerable ones, such as those presenting prodromal symptoms of psychiatric conditions. As highlighted in the present study, healthcare professionals continue to experience psychological repercussions from this extraordinary situation. Moreover, it should be noted that the current picture involves only those healthcare workers who requested an application visit. This could represent the tip of the iceberg of such a phenomenon. The hidden population of those who do not seek help should also be considered since healthcare workers with mental diseases may be more reluctant than the general population for various reasons [60], including concerns about confidentiality, potential career consequences, time constraints, and the belief that they can manage their symptoms on their own [59,61,62]. Managers and those in leading positions may play a key role as far as prevention initiatives are concerned [63].

The present study shows some limitations that warrant discussion. First, as observed in previous research [17], it is important to acknowledge the preliminary nature of the results, which were obtained through a retrospective analysis of electronic medical records using both the Infoclin and Arianna platforms. A second limitation of this study is its restriction to a single research unit since it involved only healthcare workers from a University Hospital in Northern Italy. Another limitation is the possibility that individuals in the sample may have requested their first medical assessment at a different hospital/clinical service before or during their work at the current University Hospital. The final limitation of the present study is represented by the impossibility of conducting a within-subject study, as the two groups consist of different subjects. This issue limits the inferential value of the findings, which should be taken with caution and at a descriptive level only.

## 5. Conclusions

Although a great body of literature has shown that the COVID-19 pandemic deeply affected healthcare workers’ mental health, findings from the present study underline the strong impact of the COVID-19 outbreak on the psychopathological burden in terms of an increased rate of both psychiatric comorbidities per subject and drugs prescriptions. This psychopathological burden, by causing work-related difficulties, could challenge individual health and well-being and strain the healthcare system. If these results are confirmed by further studies, taking into account that this phenomenon could be even more represented among healthcare workers, it will be of utmost importance to intervene through proactive policies and targeted interventions for frontline healthcare workers, particularly those experiencing different psychopathological comorbidities. It could be particularly helpful to conduct comprehensive and periodic screenings, considering hospital policies. This approach may facilitate not only the assessment of psychiatric burden but also the targeting of subclinical features of disease vulnerability, such as allostatic load. Indeed, by directing efforts toward subclinical symptomatology, early detection of potential issues within the hospital framework becomes feasible, and this could allow the prevention of the exacerbation of the strain on healthcare professionals’ mental health and well-being and, eventually, on the national healthcare system.

## Figures and Tables

**Table 1:** Descriptive statistics of sociodemographics of the healthcare workers requesting application visits from January 2017 to December 2022.

	Total Sample (*N* = 205)		Pre-COVID Group (*N* = 105)		COVID Group (*N* = 100)	
Variable	Mean/Number	SD/Percentage	Mean/Number	SD/Percentage	Mean/Number	SD/Percentage
Age (range: 25–69 years)	50.7	10.3	53.9	9.2	47.3	10.4
Gender						
Female	151	73.7%	78	74.3%	73	73%
Male	54	26.3%	27	25.7%	27	27%
Occupation						
Nurse	96	46.8%	51	48.6%	45	45%
Healthcare assistant	39	19%	21	20%	18	18%
Physician	15	7.3%	6	5.7%	9	9%
Radiology technician	8	3.9%	3	2.9%	5	5%
Technical assistant operator	7	3.4%	4	3.8%	3	3%
Administrative personnel	5	2.4%	4	3.8%	1	1%
Specialty trainee	5	2.4%	0	0%	5	5%
Technical operator	5	2.4%	0	0%	5	5%
Biomedical laboratory technician	4	2%	4	3.8%	0	0%
Head nurse	4	2%	2	1.9%	2	2%
Auxiliary personnel	3	1.5%	3	2.9%	0	0%
Student	3	1.5%	1	1%	2	2%
Social worker	1	0.5%	1	1%	0	0%
Chef	1	0.5%	0	0%	1	1%
Dietitian	1	0.5%	1	1%	0	0%
Physiotherapist	1	0.5%	1	1%	0	0%
Professional manager operator	1	0.5%	0	0%	1	1%
Kitchen technical operator	1	0.5%	1	1%	0	0%
Warehouse technical operator	1	0.5%	1	1%	0	0%
Receptionist	1	0.5%	1	1%	0	0%
Audiometry Technician	1	0.5%	0	0%	1	1%
Perfusion technician	1	0.5%	0	0%	1	1%
Years of employment at the hospital (range: 1–44 years)	18.9	11.1	22.5	10.5	11.2	10.6

**Table 2:** Descriptive statistics of the psychiatric diagnoses categories of the healthcare workers with at least one psychiatric diagnosis (*N* = 184) requesting application visits from January 2017 to December 2022.

Psychiatric Diagnoses Category	Number	Percentage
Mood + bipolar disorders	126	68.5%
Trauma and stressor-related disorders	23	12.5%
Anxiety disorders	17	9.2%
Substance-use disorders	5	2.7%
Personality disorders	4	2.2%
Feeding and Eating disorders	3	1.6%
Obsessive–compulsive-related disorders	2	1.1%
Sleep–wake disorders	2	1.1%
Somatic symptoms disorders	1	0.5%
Psychotic disorders	1	0.5%

**Table 3:** Descriptive statistics of the prescriptions of the healthcare workers with at least a psychiatric diagnosis (*N* = 184) requesting application visits from January 2017 to December 2022.

Prescription	Number	Percentage
Medication	132	71.7%
None	65	35.3%
Psychotherapy	8	4.3%

**Table 4:** Comparison between “pre-COVID group” and “COVID group” regarding psychiatric diagnoses categories.

Psychiatric Diagnoses Category		Pre-COVID Group (*N* = 105)	COVID Group (*N* = 100)	Total	χ^2^	*p*
Substance-use Disorders	Counting	3	2	5	0.1581	0.691
	% in Group	2.9%	2%	2.4%		
Feeding and Eating Disorders	Counting	1	2	3	0.3898	0.532
	% in Group	1%	2%	1.5%		
Anxiety Disorders	Counting	10	7	17	0.4920	0.512
	% in Group	9.5%	7%	8.3%		
Sleep–Wake Disorders	Counting	2	0	2	1.9235	0.165
	% in Group	1.9%	0%	1%		
Trauma and Stress-Related Disorders	Counting	8	15	23	2.8014	0.094
	% in Group	7.6%	15%	11.2%		
Mood + Bipolar Disorders	Counting	66	60	126	0.1765	0.674
	% in Group	62.9%	60%	61.5%		
Obsessive–Compulsive Disorders	Counting	0	2	2	2.1207	0.145
	% in Group	0%	2%	1%		
Personality Disorders	Counting	4	0	4	3.8853	0.049
	% in Group	3.8%	0%	2%		
Somatic Symptoms Disorders	Counting	1	0	1	0.9570	0.328
	% in Group	3.8%	0%	2%		
Psychotic Disorders	Counting	0	1	1	1.0551	0.304
	% in Group	0%	1%	0.5%		
None	Counting	10	11	21		
	% in Group	9.5%	11%	10.2%		

**Table 5:** Comparison between “pre-COVID group” and “COVID group” regarding rate of psychiatric comorbidities.

Group		No Diagnosis	1 Diagnosis	2+ Diagnoses	χ^2^	*p*
pre-COVID group (*N* = 105)	Counting	10	88	7	17.429	<0.001
	% in Group	9.5%	83.8%	6.7%		
COVID group (*N* = 100)	Counting	11	61	28		
	% in Group	11%	61%	28%		
TOTAL	Counting	21	149	35		
	% in Group	10.2%	72.7%	17.1%		

**Table 6:** Comparison between “pre-COVID group” and “COVID group” regarding type of prescription.

Group		Medications	None	Psychotherapy	χ^2^	*p*
pre-COVID group (*N* = 105)	Counting	59	44	2	11.508	0.003
	% in Group	56.2%	41.9%	1.9%		
COVID group (*N* = 100)	Counting	73	21	6		
	% in Group	73%	21%	6%		
TOTAL	Counting	132	65	8		
	% in Group	64.4%	31.7%	3.9%		

## Data Availability

The original data analyzed are available from the corresponding author upon reasonable request.

## Appendix A

**Table A1:** Descriptive statistics of specific psychiatric diagnoses from January 2017 to December 2022.

Psychiatric Diagnosis	Number	Percentage
Depressive disorder	53	28.8%
Anxious–depressive disorder	21	11.4%
Reactive depressive disorder	19	10.3%
Bipolar disorder	7	3.8%
Anxious disorder	7	3.8%
Adjustment disorder with depressive mood	7	3.8%
Depressive disorder—panic disorder	5	2.7%
Panic disorder	4	2.2%
Work-related stress	3	1.6%
Alcohol use disorder	3	1.6%
Anxious disorder—panic disorder	3	1.6%
Eating disorder	3	1.6%
Adjustment disorder	2	1.1%
Adjustment disorder with depressive mood and anxiety	2	1.1%
Depressive disorder—eating disorder	2	1.1%
Reactive depressive disorder—work-related stress	2	1.1%
Unspecific mood disorder	2	1.1%
Obsessive–compulsive disorder	2	1.1%
Sleep–wake disorder	2	1.1%
Post-traumatic stress disorder	2	1.1%
Suspected alcohol use disorder	2	1.1%
Work-related stress—depressive disorder	2	1.1%
Paranoid traits	2	1.1%
Agoraphobia with panic disorder	1	0.5%
Panic disorder—anxious–depressive disorder	1	0.5%
Panic disorder—sleep–wake disorder	1	0.5%
Burnout—bipolar disorder—panic disorder	1	0.5%
Burnout—work-related stress	1	0.5%
Cyclothymia	1	0.5%
Adjustment disorder with anxiety—work-related stress	1	0.5%
Anxious–depressive disorder—alcohol use disorder	1	0.5%
Anxious–depressive disorder—panic disorder	1	0.5%
Anxious–depressive disorder—sleep–wake disorder	1	0.5%
Anxious–depressive disorder—psychosomatic symptoms	1	0.5%
Anxious–depressive disorder—work-related stress	1	0.5%
Depressive disorder—alcohol use disorder	1	0.5%
Depressive disorder—eating disorder—post-partum depression	1	0.5%
Depressive disorder—obsessive–compulsive disorder—manic traits	1	0.5%
Depressive disorder—sleep–wake disorder	1	0.5%
Depressive disorder—sleep–wake disorder—anxious disorder	1	0.5%
Depressive disorder—psychosomatic symptoms	1	0.5%
Reactive depressive disorder—panic disorder	1	0.5%
Reactive depressive disorder—anxious disorder	1	0.5%
Unspecific personality disorder	1	0.5%
Obsessive–compulsive personality disorder	1	0.5%
Psychosomatic symptoms	1	0.5%
Suspected psychotic disorder	1	0.5%
Work-related stress—anxious–depressive disorder	1	0.5%
Work-related stress—depressive disorder—sleep–wake disorder	1	0.5%
Work-related stress—sleep–wake disorder	1	0.5%

## References

[1] World Health Organization Weekly Operational Update—14 December 2020. https://www.who.int/publications/m/item/weekly-epidemiological-update---14-december-2020.

[2] Tan M.Y., Rajgor D.D., Heng C.K., Chow A.S.Y., Tran A.P., Tay S.K.H. (2021). Stress and resilience of paediatric healthcare workers during COVID-19. Ann. Acad. Med. Singap..

[3] Giorgi G., Lecca L.I., Alessio F., Finstad G.L., Bondanini G., Lulli L.G., Arcangeli G., Mucci N. (2020). COVID-19-related mental health effects in the workplace: A narrative review. Int. J. Environ. Res. Public Health.

[4] Sim M.R. (2020). The COVID-19 pandemic: Major risks to healthcare and other workers on the front line. Occup. Environ. Med..

[5] Fernandez R., Sikhosana N., Green H., Halcomb E.J., Middleton R., Alananzeh I., Trakis S., Moxham L. (2021). Anxiety and depression among healthcare workers during the COVID-19 pandemic: A systematic umbrella review of the global evidence. BMJ Open.

[6] Young K.P., Kolcz D.L., O’Sullivan D.M., Ferrand J., Fried J., Robinson K. (2021). Health care workers’ mental health and quality of life during COVID-19: Results from a mid-pandemic, national survey. Psychiatr. Serv..

[7] Andhavarapu S., Yardi I., Bzhilyanskaya V., Lurie T., Bhinder M., Patel P., Pourmand A., Tranet Q.K. (2022). Post-traumatic stress in healthcare workers during the COVID-19 pandemic: A systematic review and meta-analysis. Psychiatry Res..

[8] Shanafelt T., Ripp J., Trockel M. (2020). Understanding and addressing sources of anxiety among health care professionals during the COVID-19 pandemic. JAMA.

[9] Stelnicki A.M., Carleton R.N., Reichert C. (2020). Nurses’ mental health and well-being: COVID-19 impacts. Can. J. Nurs. Res..

[10] Greenberg N., Docherty M., Gnanapragasam S., Wessely S. (2020). Managing mental health challenges faced by healthcare workers during COVID-19 pandemic. BMJ.

[11] Petzold M.B., Plag J., Ströhle A. (2020). Umgang mit psychischer Belastung bei Gesundheitsfachkräften im Rahmen der COVID-19-Pandemie. Nervenarzt.

[12] Lasalvia A., Bonetto C., Porru S., Carta A., Tardivo S., Bovo C., Ruggeri M., Amaddeo F. (2021). Psychological impact of COVID-19 pandemic on healthcare workers in a highly burdened area of north-east Italy. Epidemiol. Psychiatr. Sci..

[13] Marinaccio A., Brusco A., Bucciarelli A., D’Amario S., Iavicoli S. (2021). Temporal trend in the compensation claim applications for work-related COVID-19 in Italy. Med. Lav..

[14] De Matteis S., Pira E., Mutti A. (2021). The COVID-19 pandemic and occupational medicine: Impact and opportunities. Med. Lav..

[15] Sahar Kostis Y.O., Rinsky-Halivni L., Cohen C., Zack O., Dekel R., Moshe S. (2021). Fitness for work during the COVID-19 disease: Principles and suggested assistive tool for protecting workers during the pandemic era. Int. Arch. Occup. Environ. Health.

[16] Mendola M., Leoni M., Cozzi Y., Manzari A., Tonelli F., Metruccio F., Tosti L., Battini V., Cucchi I., Costa M.C. (2022). Long-term COVID symptoms, work ability and fitness to work in healthcare workers hospitalized for Sars-CoV-2 infection. Med. Lav..

[17] Gostoli S., Nicolucci L., Malaguti C., Patierno C., Carrozzino D., Balducci C., Zaniboni S., Lodi V., Petio C., Rafanelli C. (2022). Mental illness and work-related limitations in healthcare workers: A preliminary retrospective study. Int. J. Environ. Res. Public Health.

[18] American Psychiatric Association (2013). Diagnostic and Statistical Manual of Mental Disorders.

[19] World Medical Association (2013). World medical association declaration of Helsinki: Ethical principles for medical research involving human subjects. JAMA.

[20] Almeida M., Fletcher S. (2022). Serious mental illness in women. Curr. Opin. Psychiatry.

[21] Wittchen H.U., Jacobi F., Rehm J., Gustavsson A., Svensson M., Jönsson B., Olesen J., Allgulander C., Alonso J., Faravelli C. (2011). The size and burden of mental disorders and other disorders of the brain in Europe 2010. Eur. Neuropsychopharmacol..

[22] Seedat S., Scott K.M., Angermeyer M.C., Berglund P., Bromet E.J., Brugha T.S., Demyttenaere K., de Girolamo G., Haro J.M., Jin R. (2009). Cross-national associations between gender and mental disorders in the World Health Organization World Mental Health Surveys. Arch. Gen. Psychiatry.

[23] Tedstone Doherty D., Kartalova-O’Doherty Y. (2010). Gender and self-reported mental health problems: Predictors of help seeking from a general practitioner. Br. J. Health Psychol..

[24] Khamisa N., Peltzer K., Ilic D., Oldenburg B. (2016). Work related stress, burnout, job satisfaction and general health of nurses: A follow-up study: WRS, BO, JS and GH of nurses: Follow-up study. Int. J. Nurs. Pract..

[25] Stelnicki A.M., Jamshidi L., Angehrn A., Hadjistavropoulos H.D., Carleton R.N. (2021). Associations between burnout and mental disorder symptoms among nurses in Canada. Can. J. Nurs. Res..

[26] Bellotti L., Zaniboni S., Balducci C., Grote G. (2021). Rapid review on COVID-19, work-related aspects, and age differences. Int. J. Environ. Res. Public Health.

[27] Tan B.Y.Q., Chew N.W.S., Lee G.K.H., Jing M., Goh Y., Yeo L.L.L., Zhang K., Chin H.-K., Ahmad A., Ahmed Khan F. (2020). Psychological impact of the COVID-19 pandemic on health care workers in Singapore. Ann. Intern. Med..

[28] Al-Hanawi M.K., Mwale M.L., Alshareef N., Qattan A.M.N., Angawi K., Almubark R., Alsharqi O. (2020). Psychological distress amongst health workers and the general public during the COVID-19 pandemic in Saudi Arabia. Risk Manag. Healthc. Policy.

[29] Walton M., Murray E., Christian M.D. (2020). Mental health care for medical staff and affiliated healthcare workers during the COVID-19 pandemic. Eur. Heart J. Acute. Cardiovasc. Care.

[30] Chiang Y.-M., Chang Y. (2012). Stress, depression, and intention to leave among nurses in different medical units: Implications for healthcare management/nursing practice. Health Policy.

[31] Tsay S.-F., Wang H.-H. (2007). The making and development of policy concerning nurse practitioners in Taiwan. Hu Li Za Zhi.

[32] Hayes L.J., O’Brien-Pallas L., Duffield C., Shamian J., Buchan J., Hughes F., Spence Laschinger H.K., North N. (2012). Nurse turnover: A literature review—An update. Int. J. Nurs. Stud..

[33] Cheung T., Yip P.S.F. (2015). Depression, anxiety and symptoms of stress among Hong Kong nurses: A cross-sectional study. Int. J. Environ. Res. Public Health.

[34] Maharaj S., Lees T., Lal S. (2018). Prevalence and risk factors of depression, anxiety, and stress in a cohort of Australian nurses. Int. J. Environ. Res. Public Health.

[35] Preti E., Di Mattei V., Perego G., Ferrari F., Mazzetti M., Taranto P., Di Pierro R., Madeddu F., Calati R. (2020). The psychological impact of epidemic and pandemic outbreaks on healthcare workers: Rapid review of the evidence. Curr. Psychiatry Rep..

[36] Spector P.E. (1997). Job Satisfaction: Application, Assessment, Causes and Consequences.

[37] McEwen B.S., Stellar E. (1993). Stress and the individual: Mechanisms leading to disease. Arch. Intern. Med..

[38] McEwen B.S. (1998). Adaptation, and disease: Allostasis and allostatic load. Ann. N. Y. Acad. Sci..

[39] McEwen B.S. (2007). Physiology and neurobiology of stress and adaptation: Central role of the brain. Physiol. Rev..

[40] McEwen B.S., Wingfield J.C. (2003). The concept of allostasis in biology and biomedicine. Horm. Behav..

[41] Fava G.A., McEwen B.S., Guidi J., Gostoli S., Offidani E., Sonino N. (2019). Clinical characterization of allostatic overload. Psychoneuroendocrinology.

[42] Juster R.-P., Marin M.-F., Sindi S., Nair N.P.V., Ng Y.K., Pruessner J.C., Lupien S.J. (2011). Allostatic load associations to acute, 3-year and 6-year prospective depressive symptoms in healthy older adults. Physiol. Behav..

[43] Juster R.-P., Sasseville M., Giguère C.-É., Consortium S., Lupien S.J. (2018). Elevated allostatic load in individuals presenting at psychiatric emergency services. J. Psychosom. Res..

[44] Tomba E., Offidani E. (2012). A clinimetric evaluation of allostatic overload in the general population. Psychother. Psychosom..

[45] Sun J., Wang S., Zhang J.-Q., Li W. (2007). Assessing the cumulative effects of stress: The association between job stress and allostatic load in a large sample of Chinese employees. Work Stress.

[46] Alonso J., Vilagut G., Mortier P., Ferrer M., Alayo I., Aragón-Peña A., Aragonès E., Campos M., Cura-González I.D., Emparanza J.I. (2021). Mental health impact of the first wave of COVID-19 pandemic on Spanish healthcare workers: A large cross-sectional survey. Rev. Psiquiatr. Salud. Ment..

[47] Tran T.V., Nguyen H.C., Pham L.V., Nguyen M.H., Nguyen H.C., Ha T.H., Phan D.T., Dao H.K., Nguyen P.B., Trinh M.V. (2020). Impacts and interactions of COVID-19 response involvement, health-related behaviours, health literacy on anxiety, depression and health-related quality of life among healthcare workers: A cross-sectional study. BMJ Open.

[48] Mattila E., Peltokoski J., Neva M.H., Kaunonen M., Helminen M., Parkkila A.K. (2020). COVID-19: Anxiety among hospital staff and associated factors. Ann. Med..

[49] Lin Y.E., Tseng C.N., Wang M.F., Wu S.F.V., Jane S.W., Chien L.Y. (2020). Anxiety and work stress among newly employed nurses during the first year of a residency programme: A longitudinal study. J Nurs. Manag..

[50] Duarte D., El-Hagrassy M.M., Couto T., Gurgel W., Frey B.N., Kapczinski F., Corrêa H. (2022). Physician suicide demographics and the COVID-19 pandemic. Braz. J. Psychiatry.

[51] Duarte D., El-Hagarassy M.M., Couto T., Gurgel W., Minuzzi L., Saperson K. (2023). Challenges and potential solutions for physician’s suicide risk factors in the covid-19 era: Psychiatric comorbidities. Medicine judicialization, and burnout. Trends Psychiatry Psychother..

[52] Duarte D., El-Hagrassy M.M., Couto T.C.E., Gurgel W., Fregni F., Corrêa H. (2020). Male and female physician suicidality: A systematic review and meta-analysis. JAMA Psychiatry.

[53] Gold K.J., Sen A., Schwenk T.L. (2013). Details on suicide among US physicians: Data from the National Violent Death Reporting System. Gen. Hosp. Psychiatry.

[54] Iannelli R.J., Finlayson A., Brown K.P., Neufeld R., Gray R., Dietrich M.S. (2014). Suicidal behavior among physicians referred for fitness-for-duty evaluation. Gen. Hosp. Psychiatry.

[55] Yeung K.S., Hernandez M., Mao J.J., Haviland I., Gubili J. (2018). Herbal medicine for depression and anxiety: A systematic review with assessment of potential psycho-oncologic relevance. Phytother. Res..

[56] Brunette M.F., Noordsy D.L., Xie H., Drake R.E. (2003). Benzodiazepine use and abuse among patients with severe mental illness and co-occurring substance use disorders. Psychiatr. Serv..

[57] Johnson M.R., Lydiard R.B. (1998). Comorbidity of major depression and panic disorder. J. Clin. Psychol..

[58] Pereira-Lima K., Mata D.A., Loureiro S.R., Crippa J.A., Bolsoni L.M., Sen S. (2019). Association between physician depressive symptoms and medical errors: A systematic review and meta-analysis. JAMA Netw. Open.

[59] Harvey S.B., Epstein R.M., Glozier N., Petrie K., Strudwick J., Gayed A., Dean K., Henderson M. (2021). Mental illness and suicide among physicians. Lancet.

[60] Hawton K., Malmberg A., Simkin S. (2004). Suicide in doctors. A psychological autopsy study. J. Psychosom. Res..

[61] Wu F., Ireland M., Hafekost K., Lawrence D. National Mental Health Survey of Doctors and Medical Students. Beyond Blue. https://espace.curtin.edu.au/bitstream/handle/20.500.11937/90008/89832.pdf?sequence=2.

[62] Dyrbye L.N., West C.P., Sinsky C.A., Goeders L.E., Satele D.V., Shanafelt T.D. (2017). Medical licensure questions and physician reluctance to seek care for mental health conditions. Mayo Clin. Proc..

[63] Toderi S., Balducci C. (2018). Stress-preventive Management Competencies, psychosocial work environments, and affective well-being: A multilevel, multisource investigation. Int. J. Environ. Res. Public Health.

